# Biomarkers in Rare Demyelinating Disease of the Central Nervous System

**DOI:** 10.3390/ijms21218409

**Published:** 2020-11-09

**Authors:** Marina Boziki, Styliani-Aggeliki Sintila, Panagiotis Ioannidis, Nikolaos Grigoriadis

**Affiliations:** 2nd Neurological University Department, Aristotle University of Thessaloniki, AHEPA General Hospital, 54634 Thessaloniki, Greece; bozikim@auth.gr (Μ.Β.); lina17puma@yahoo.gr (S.-A.S.); ispanagi@auth.gr (P.I.)

**Keywords:** rare neurological diseases, multiple sclerosis, acute disseminated encephalomyelitis, neuromyelitis optica spectrum disorders, myelin oligodendrocyte glycoprotein antibody disease, neuroinflammation

## Abstract

Rare neurological diseases are a heterogeneous group corresponding approximately to 50% of all rare diseases. Neurologists are among the main specialists involved in their diagnostic investigation. At the moment, a consensus guideline on which neurologists may base clinical suspicion is not available. Moreover, neurologists need guidance with respect to screening investigations that may be performed. In this respect, biomarker research has emerged as a particularly active field due to its potential applications in clinical practice. With respect to autoimmune demyelinating diseases of the Central Nervous System (CNS), although these diseases occur in the frame of organ-specific autoimmunity, pathology of the disease itself is orchestrated among several anatomical and functional compartments. The differential diagnosis is broad and includes, but is not limited to, rare neurological diseases. Multiple Sclerosis (MS) needs to be differentially diagnosed from rare MS variants, Acute Disseminated Encephalomyelitis (ADEM), the range of Neuromyelitis Optica Spectrum Disorders (NMOSDs), Myelin Oligodendrocyte Glycoprotein (MOG) antibody disease and other systemic inflammatory diseases. Diagnostic biomarkers may facilitate timely diagnosis and proper disease management, preventing disease exacerbation due to misdiagnosis and false treatment. In this review, we will describe advances in biomarker research with respect to rare neuroinflammatory disease of the CNS.

## 1. Rare Disorders’ Classifications

According to the European Regulation on Orphan Medicinal Products, a rare disease is a disease that affects less than five in 10,000 persons in Europe [1]. Rare diseases are classified in the Orphanet, a database launched in order to assist clinicians and patients in navigating relevant symptoms and medical resources [2]. Accordingly, the Orphanet nomenclature is under constant development as a medical terminology specific for rare diseases, syndromes and clinical entities that have been described and registered [3]. In the Orphanet database, several classification approaches are followed, the most prominent depending on the symptoms. According to this paradigm, a clinical entity, especially the ones with multi-organ involvement, may be found under several classifications [4].

## 2. Rare Neurological Diseases

Rare neurological diseases are a highly heterogeneous group corresponding approximately to 50% of all rare diseases [5]. However, this estimation includes not only primary neurological disease but also several systemic multi-organ diseases associated partly with neurologic manifestations. Due to the complex disease phenotypes that often include neurological manifestations as presenting symptoms, neurologists are among the main specialists involved in diagnostic investigation [6]. However, the rarity of the described clinical entities poses difficulty in diagnosis due to the lack of experience of most neurologists. This fact underlines a need for highly specialized neurologists serving in reference centres [7].

Reference centres need to function as centres of excellence for neurological rare diseases, primarily operating in a network setting on a national and/or international level, dedicated to diagnosing and registering patients [8]. Moreover, reference centres are expected to conduct research with respect to thorough disease phenotyping, the exploration of disease mechanism and relevant biomarkers, and the assessment of the efficacy of newly available treatments in a multi-centre setting [9].

However, the need for increased awareness and alertness on behalf of neurologists in order to refer the patient to a highly specialized centre remains [6]. At the moment, consensus guidelines on which neurologists may base clinical suspicion are not available. Moreover, neurologists need guidance with respect to screening investigations that may be performed, thus saving valuable time and financial resources for the patient and the health system [6]. As a general rule, syndromic and/or multisystem involvement should prompt diagnostic investigation relevant to a rare neurological disease. For example, a combination of diabetes mellitus and sensorineural hearing loss with onset in young adult age should strongly indicate the need for a test in search of mitochondrial disease [10].

## 3. In Search for an “Ideal” Biomarker

Biomarker research has emerged as a particularly active research field due to its potential applications in clinical practice with respect to disease diagnostics and prognostic evaluation [11]. Ideally, a biomarker constitutes a characteristic that can be objectively and easily measured and evaluated. A biomarker may indicate aspects of a biological process in health and/or upon pathology or as a response to a pharmacological treatment [12]. Biomarkers that are highly likely to be applied in clinical practice are acquired via procedures safe for the patient; are therefore minimally invasive; and are evaluated on the basis of a relatively low-cost, easily conducted and standardized method [13]. Based on the clinical feature that they depict, biomarkers are usually categorized as predictive (identifying risk of disease), diagnostic (distinguishing between health and disease), monitoring (indicating activity/remission/progression/improvement or worsening) and safety biomarkers (usually related to a pharmacological treatment) [14]. With respect to autoimmune demyelinating disease of the Central Nervous System (CNS), a major issue arises when considering biomarker investigation: although these diseases occur in the frame of organ-specific autoimmunity, the pathology of the disease itself is orchestrated among several anatomical and functional compartments of the human body [15]. The immune system and the CNS participate in a bidirectional communication, and triggering of immune responses may occur in the periphery in organs remote to the CNS [16]. This fact subjects the immune mechanisms to several “checkpoints” of regulation, as several cell types are influenced and participate in the process that leads in neuroinflammation (i.e., T-cell, B-cell and myeloid cells in the periphery and microglia, astrocytes, infiltrating lymphocytes and/or macrophages in the CNS) [17]. Moreover, this diversity may, at least in part, contribute in the considerable intraindividual heterogeneity that is evident in autoimmune disease of the CNS [18]. Recently, the technological advances that allow for a comprehensive “omic” fingerprint analysis (i.e., genomic, metabolomic, proteomic, transcriptomic, epigenomic and others) led to the generation of large datasets that, combined with an accurate phenotypic classification, may elucidate novel disease pathways and may indicate relevant biomarker candidates [19].

An example of such a biomarker, based on sequencing technology and currently under investigation for neurodegenerative and/or neuroinflammatory disease, is several types of noncoding RNAs that are universally considered as molecular markers of epigenetic alterations as well as of regulation of gene expression at the transcriptional and posttranscriptional levels. As noncoding RNAs participate in cell proliferation and differentiation in the CNS [20] as well as in synaptic plasticity [21], they are regarded as factors potentially associated with several neurodegenerative diseases, such as Alzheimer’s Disease [22], Parkinson’s Disease [23] and Huntington’s Disease [24]. In the field of rare genetic diseases, analysis of microRNAs and long noncoding RNAs is an emerging promising strategy that is expected to serve molecular diagnosis and therapy [25]. Noncoding RNAs have also been implicated in the diagnosis and phenotyping of autoimmune inflammatory CNS disease. MicroRNAs (miRNA) are noncoding RNAs which are composed only of 20–22 nucleotides. They act as negative regulators of gene expression at a posttranscriptional level [26] and may serve as diagnostic biomarkers for a variety of neurological diseases [27], including MS [28] (reviewed in [29]). Blood miRNAs may function as circulating biomarkers of the disease [30] and may also be an indicator of Relapsing-Remitting MS (RRMS) when examined in peripheral blood leukocytes [31]. Specific miRNAs have been associated with disability and disease activity [32,33], whereas others have been related to progressive forms of the disease [34]. In addition, Cerebrospinal Fluid (CSF) miR150 may serve as a biomarker for MS as well as CIS at risk towards MS conversion [35,36]. Other noncoding RNAs, such as Long noncoding RNAs (LncRNAs), have been proposed to be involved in neurodegenerative and/or demyelinating diseases of the CNS. LncRNAs have heterogeneous linear RNA transcripts of lengths that exceed 200 nucleotides. LncRNAs appear to play a role in multiple cellular pathways, such as immune system control, and survival and proliferation of cancer cells. In CNS tissues involved in neurodegenerative processes, LncRNAs were shown to act as molecular markers of pathogenic processes when studied in models of oxidative stress [37,38,39]. Moreover, abnormal expression of LncRNAs may contribute to the development of MS by influencing immunological pathways [40,41]. The relation between LncRNAs and MS is further confirmed by genome-wide scale studies conducted in blood and CNS tissue [42]. In this respect, LncRNAs may function as prognostic biomarkers in MS [43].

The differential diagnosis of neuroinflammatory disease of the CNS is broad and includes, but is not limited to, rare neurological diseases [44]. MS needs to be differentially diagnosed from rare MS variants; Acute Disseminated Encephalomyelitis (ADEM); the range of Neuromyelitis Optica Spectrum Disorders (NMOSDs); Myelin Oligodendrocyte Glycoprotein (MOG) antibody disease; other inflammatory diseases such as primary angiitis of the CNS, systemic lupus erythematosus, Behcet’s disease and neurosarcoidosis; several infectious diseases of the CNS such as Lyme neuroborreliosis and viral CNS-associated diseases; and neoplastic pathologies such as primary CNS lymphoma and solid tumours [45]. Moreover, several rare neurological diseases of neurometabolic origin, either hereditary or with strong genetic predisposition (Leber hereditary optic neuropathy, Leigh syndrome, Kearns–Sayre syndrome, biotin-responsive basal ganglia disease, acute necrotizing encephalopathy and various leukodystrophies), often mimic neuroinflammatory diseases of the CNS [46]. 

Diagnostic biomarkers, where available, are of particular value in neuroinflammatory disease of the CNS because they may facilitate timely diagnosis and proper disease management, preventing disease exacerbation due to misdiagnosis and false treatment [47].

## 4. Orphanet Classification of Rare Neurological Diseases

In the Orphanet classification of rare neurological diseases, rare neuroinflammatory or neuroimmunological disease is mentioned under ORPHA code 182064 [48].

Of those, CNS neuroinflammatory disease is collectively mentioned under the “umbrella” term MS variant (ORPHA:228145). However, of the clinical entities described, only Baló Concentric Sclerosis (BCS) (ORPHA:228165), Marburg acute multiple sclerosis (ORPHA:228157) and Schilder’s disease (ORPHA:59298) are universally considered rare variants of MS [49].

ADEM (ORPHA:83597) and Neuromyelitis Optica (NMO) (ORPHA:71211) are distinct clinical entities, and paediatric MS (ORPHA:477738) is diagnosed on the basis of the 2017 revised McDonald criteria with early age at onset (prior to 17 years of age) [50].

In this review, we will describe recent advances in the field of biomarker research with respect to rare neuroinflammatory diseases of the CNS. Due to the recent description of anti-MOG antibody-mediated disease (MOGAD), this rare clinical entity defined by a distinct biomarker is also included.

## 5. Multiple Sclerosis Variants

The so-called variants of MS are rare, clinically important presentations of MS, as they cause considerable diagnostic uncertainty and, sometimes, misdiagnosis [49]. Each is characterized by unusual imaging presentation, often posing dilemmas of differential diagnosis from CNS tumours and/or other space-occupying lesions [51]. Due to their unusual imaging presentation and overall worse prognosis as well as the fact that these variants typically are not responsive to the classical Disease Modifying Treatments (DMTs) for MS, it has been suggested that MS variants may in fact constitute a disease group distinct from MS [52]. As these patients may undergo brain biopsy for diagnostic reasons, this assumption is further supported by neuropathological analyses available in this patient group. However, the rarity of these cases and the significant clinical and radiological heterogeneity do not allow for safe conclusion so far [49]. The currently clinically most important variants (most likely to be seen or considered in clinical practice) include BCS, Marburg MS and Schilder’s disease [53].

### 5.1. Baló Concentric Sclerosis

BCS is regarded as a rare variant of MS [54]. It characteristically occurs as a discrete concentrically layered lesion in cerebral white matter. This distinctive appearance helps to distinguish it from the lesions of conventional MS and from tumefactive demyelinating lesions [55]. The median age of onset of BCS is 34 years, showing a female to male ratio of about 2:1. BCS is more common in patients of east Asian origin [52]. Commonly reported manifestations are similar to that of intracerebral mass lesion, such as nausea, vomiting, severe headache, encephalopathy and/or confused state, especially in cases with associated prominent mass effect and/or edema on brain MRI [53,56,57]. Occasionally, clinical presentation is more acute, mimicking stroke. In rare cases, lesions may be asymptomatic [55]. Differential diagnoses include brain tumours, such as glioblastoma multiforme or primary CNS lymphoma, infarction, brain abscess and tumefactive demyelination [55].

BCS is in essence a diagnosis based on the radiological appearance of the lesions [55]. On brain Magnetic Resonance Imaging (MRI), T1-weighted imaging characteristically shows alternating isointense and hypointense concentric rings. On T2-weighted sequences, hyperintense lamellae surround a centre of T2 hyperintensity. Lesion oedema may frequently occur. Gadolinium enhancement occurs more frequently at the periphery of the lesion [53]. Lesions are located predominantly in the cerebral white matter, sparing cortical U-fibres. Other sites include basal ganglia, pons and cerebellum [55]. Baló lesions can be multiple at onset or can appear as solitary lesions. Macroscopically, lesions are usually larger than typical MS lesions [55].

Pathologically, Baló lesions are well described and typically consist of cerebral white matter oligodendrocyte loss and demyelination, sparing the cortical grey matter. Their alternating ring appearance is due to areas of relative myelin preservation and myelin loss [54].

In spite of the characteristic morphology of the lesions on MRI and in pathology, Baló disease is typically considered an MS variant due to a significant clinical overlap with conventional MS [54]. Moreover, Baló-like lesions may occur during the course of RRMS (at least 55% of patients have typical MS lesions elsewhere on their MRI scan) [53]. The presence of Oligoclonal Bands (OCBs) in the CSF in patients with Baló lesions in the MRI upon the first demyelinating episode has been associated with a typical MS clinical course [55]. Based on these observations, corticosteroids and first-line DMTs are recommended as an appropriate first step [54]. Plasma exchange has been suggested as a reasonable second-line option, often recommended as rescue therapy, especially in tumefactive demyelinating lesions [55]. Classic immune-suppressive agents may also be considered in the frame of a highly active disease associated with rapid disability progression [55]. The prognosis varies from complete clinical and radiological recovery to substantial morbidity or even death [54].

### 5.2. Marburg Variant

Marburg disease was first described as a variant of MS in 1906 and may be best regarded as an extreme end of the spectrum of inflammatory demyelinating disorders of the CNS [58]. It is an acute monophasic form of MS, characterized by rapid onset and progression of the demyelination process, leading to death usually within a year [58]. Histologically, there is severe myelin and axon destruction and prominent tissue necrosis [58]. Unlike MS, lesions tend to have more inflammatory infiltrates and to be more destructive [59]. Radiologically, they occur simultaneously in all affected areas, either hemispheres or brainstem [59]. If it is presented as a sole big lesion, it should be differentiated from a brain tumour or abscess [59]. They usually show significant mass effect and edema and may resemble the lesions in tumefactive MS or ADEM [58]. The diagnosis is usually made retrospectively, based on the described disease course, whereas a more chronic remitting course following the initial presentation of high activity signifies a final diagnosis of MS [59]. Management of an acute episode for this fulminant MS variant includes early administration of intravenous corticosteroids. In cases not responding to steroids, plasma exchange is beneficial [58]. Early immunosuppressive therapy, such as cyclophosphamide and mitoxantrone, should be considered if deficits are severe and/or progress despite steroids and plasma exchange [58].

### 5.3. Schilder’s Disease

Myelinoclastic Diffuse Sclerosis (MDS), also termed Schilder’s disease or encephalitis periaxialis diffusa, is a rare inflammatory demyelinating disorder of the CNS. It was first described in 1912 by the Austrian psychiatrist Paul Ferdinand Schilder [60]. MDS and MS are immunopathologically distinct entities in most instances [60]. There is a low frequency of OCBs in MDS, which is in contrast to MS [61]. CSF pleocytosis is usually absent in the majority of patients with MDS, and CSF total protein levels are often higher than in typical MS [60]. MDS is characterized by one or two extensive, often bilateral and symmetrical demyelinating white matter lesions in the centrum semiovale [62] with frequent contrast enhancement [58]. The spinal cord and the cerebral cortex seem to be mostly spared in MDS [60]. Many experts have abandoned the concept of Schilder’s disease due to early series with cases of adrenoleukodystrophy [58]. In terms of management, several case reports suggest that (high-dose) steroid treatment may be beneficial [63]. Immunomodulatory drugs approved for the treatment of MS may be used in selected patients (OCB-positive and in borderline cases suggestive of either MDS or MS). More data are needed before definite recommendations on the treatment of MDS can be made [60].

### 5.4. Paediatric-Onset Multiple Sclerosis

Paediatric-onset MS (POMS) is a diagnosis attributed to children and adolescents with disease onset prior to 18 years of age [64]. Τhe diagnostic criteria for the paediatric population differ from those for adults, particularly in children aged < 11 years [64]. In this particular paediatric population, the clinical presentation of MS frequently is atypical compared to the typically described disease in adults [65]. Moreover, postinfectious and post-vaccinal demyelinating events are frequently observed in children and are commonly associated with ADEM [64]. ADEM is a monophasic disease. However, in few patients with ADEM, a second episode either with clinical and radiological presentation similar to the first episode (recurring ADEM) or a second demyelinating episode resembling MS may occur [65]. In the latter scenario, diagnosis of paediatric MS may be applied, provided that the clinical presentation is not suggestive of encephalopathy and that the event is accompanied by new lesions on an MRI that are suggestive of MS [65]. In this respect, several cases of ADEM in children may progress into developing POMS and the differential diagnosis from recurring ADEM requires full diagnostic evaluation in the absence of a definite diagnostic biomarker [64]. The presence of OCBs in the CSF after the first clinical demyelinating attack is associated with a high risk of developing MS [65]. However, the substitution of Dissemination In Time (DIT) criteria with the presence of OCBs in CSF has to be validated in large prospective cohorts of the paediatric population [64].

POMS manifests distinctive features compared to adult-onset MS; however, the age of onset remains a defining characteristic. In POMS, the gender ratio differs across the age of onset, with female predominance starting after puberty [65]. Children aged >10 years are at a higher risk of MS diagnosis after their first clinical episode [64]. POMS is usually an RRMS, with higher activity compared to adult-onset MS in terms of annualized relapse rate and median time intervals between the first and the second clinical attack [66]. Moreover, relapses tend to be more severe in children compared to the adult patients, although with better recovery [64]. POMS appears to involve the cerebellum and the brainstem more frequently, and this fact may account for the overall worse prognosis of POMS compared to adult-onset MS [64]. Due to the considerable high activity of the disease upon presentation and the earlier accumulation of disability in this population, fingolimod, a DMT indicated for highly active MS, was recently approved for POMS in addition to interferon-β, a DMT typically prescribed as a first-line agent [64].

### 5.5. Biomarkers for Rare MS Variants

The application of OCBs and the Immunoglobulin G (IgG) index as a biomarker diagnostic for MS in patients with Baló lesions in the MRI is subjected to the consensus of the 2017 revised McDonald criteria for RRMS. However, it is noteworthy that, overall, the frequency of OCBs and the IgG index in Baló disease is low compared to MS [67]. This pattern more closely resembles the NMOSD and MOGAD diseases for which the role of autoantibodies and the outside-in paradigm in the pathogenesis of neuroinflammation have been recently well-described [68]. This observation, together with the fact that patients with Baló disease seem to respond more effectively to B-cell targeting management approaches, such as plasma exchange, as do other patients with overall tumefactive presentation on brain MRI, suggest that, at least in a percentage of patients, Baló lesions may indicate an underlying pathology immunologically distinct from MS [67].

Children with a first demyelinating episode in the frame of neuroinflammation may be clinically presented as ADEM, MS (in which case, it is defined as POMS), NMOSD or the newly described MOGAD [69]. Epidemiological evidence indicates that ADEM is the main clinical entity to differentially diagnose from POMS [65]. The frequent presence of anti-MOG antibodies in children with clinical presentation of ADEM dictates that practically all children with an ADEM-like episode are tested for anti-MOG antibodies and that seropositivity essentially verifies MOGAD diagnosis [70].

Overall, MS variants are associated with a severe course with symptoms frequently indicating a cerebral tumour, such as a variable degree of loss of consciousness; signs of intracranial hypertension; seizures; and neuropsychological deficits as aphasia, apraxia and hemineglect [58]. Due to this fact, a significant percentage of the patients with a rare MS variant have been diagnosed on the basis of an invasive approach. Neuroradiological features that indicate demyelination in the context of an MS variant have been proposed [62], but a universal approach is still lacking, possibly due to the rarity and the clinical and radiological heterogeneity of the cases [69]. In line with these observations, rare MS variants remain primarily a radiological diagnosis following exclusion of a tumour, either by MR-spectroscopy or, in cases where radiology is not conclusive, by CNS biopsy [52]. A thorough dissection of the underlying pathology is therefore necessary in order to elucidate candidate biomarkers of diagnostic value [53].

## 6. ADEM

Monophasic ADEM is a rare autoimmune demyelinating disorder of the CNS defined as a single multifocal demyelinating event associated with encephalopathy, not explained by fever [70]. It is more frequent during childhood, whereas in adults, the age of presentation is between 33 and 41 years [70]. In the majority of cases, onset is preceded by viral/bacterial infection or active immunization several weeks before disease onset [58]. The prevailing hypothesis is that of molecular mimicry, according to which CNS autoimmunity is triggered by a viral or bacterial microorganism with shared similarity to myelin autoantigens [70]. Typical MRI findings are large, poorly defined hyperintense lesions in cerebral white and grey matter. Deep grey matter lesions can also be present [58]. White matter involvement is typically bilateral and asymmetric [49]. The classic form of ADEM (70–90% of all cases) is characterized by a monophasic disease course. However, variable proportions of patients (10–30% of all cases) present with a multiphasic course ADEM [70]. ADEM variants are acute haemorrhagic leukoencephalitis (an extremely severe, hyperacute variant of ADEM with rapid progression to coma and death from massive brain edema), recurrent unilateral or bilateral optic neuritis, acute transverse myelitis (common manifestation of postinfectious or post-vaccinal ADEM), and combined central and peripheral involvement of the nervous system (patients are significantly older and have a worse prognosis) [70]. At the onset of disease, intravenous methylprednisolone might be associated with IV acyclovir, if acute viral encephalitis is suspected [70]. IV immunoglobulin (IVIG) treatment is regarded as a second-line treatment reserved for cases unresponsive to steroids [71]. Plasma exchange is recommended in refractory patients with fulminant disease [72]. About 65–85% of paediatric ADEM patients have a favourable prognosis, most of whom recover completely within few weeks [73]. Adult patients frequently show residual focal motor deficits or epileptic episodes and higher rates of hospitalization and mortality [70].

In ADEM, CSF examination may show increased protein and mild pleocytosis (lymphocytes and monocytes) [58]. Intrathecal OCBs are rare, and when detected, they tend to manifest as a “mirror” pattern (similar to seruma and the CSF; therefore, antibody production is not intrathecally restricted) [74]. According to the current diagnostic criteria, ADEM remains a diagnosis of exclusion; therefore, no indicative diagnostic biomarker exists [70].

## 7. Neuromyelitis Optica Spectrum Disorder (NMOSD) vs. Anti-MOG (+) Disease (MOGAD)

NMOSD and MOGAD are both immune-mediated inflammatory conditions of the CNS that frequently involve optic nerves, the spinal cord and major mimics of MS [75]. However, NMOSD and MOGAD are clearly distinct from MS, as indicated by differences in disease mechanism and histopathology [76]. For these clinical entities, the presence of a diagnostic biomarker indicates the mediated pathology and excludes alternative diagnoses: anti-aquaporin-4 (anti-AQP4) and anti-MOG antibodies for NMOSD and MOGAD, respectively [75]. The antigen targeted by these antibodies determines the underlying pathology, that is, astrocytopathy for NMOSD and oligodendropathy for MOGAD [77,78].

### 7.1. Neuromyelitis Optica Spectrum Disorder (NMOSD)

NMO and NMOSD collectively refer to a rare antibody-mediated disease of the CNS. The term was first described by Eugène Devic and his student Fernand Gault in 1894 [79]. Since 2004, when the aquaporin-4 (AQP-4) water channel was identified as the antigen target of the antibodies that mediated the disease, NMO was distanced from MS on the basis of a diagnostic biomarker [80]. However, not all patients clinically resembling NMO are anti-AQP4 seropositive; however, these patients may otherwise fulfil the clinical and/or radiological criteria for NMOSD diagnosis. Moreover, according to the 2015 criteria for the diagnosis of NMOSD, the presence of anti-MOG antibodies accounts for a small percentage of patients that clinically resemble NMOSD and are, therefore, collectively classified under this term [81]. Over the last years, however, it became increasingly accepted that the presence of anti-MOG antibodies signifies a distinct pathology and, therefore, a different clinical entity (MOGAD) from NMOSD in spite of the clinical overlap in some patients [81]. Moreover, patients with MOGAD also exhibit considerable clinical heterogeneity, including presentations other than NMOSD [82]. This fact underlines the definition of NMOSD as a clinical spectrum of disease that is clinically heterogenous and, with respect to the double-seronegative patients, that is, anti-AQP4 and anti-MOG seronegative, has possibly a considerable yet poorly investigated, so far, pathological heterogeneity [83]. In contrast to MOGAD, where patients are by definition anti-MOG seropositive, not all patients with NMOSD are AQP4-seropositive [68]. The fact that anti-AQP4 antibodies evidently mediate disease in patients with NMOSD poses considerable question; whether another distinct diagnostic biomarker remains to be elucidated for this particular group of patients [68].

AQP4 is a major water channel in the CNS, exhibiting increased abundance in specific areas of the brainstem and the optic nerves. AQP4 is expressed in the processes of astrocytes that participate in the formation of the blood–brain barrier [84,85]. AQP4 is also expressed outside the CNS, in the kidney, stomach, airway tract, secretory glands and skeletal musculature. However, the presence of anti-AQP4 antibodies is not associated with organ-related pathology outside the CNS [79]. Rarely, anti-AQP4 antibodies have been described in patients with myasthenia gravis but the clinical significance of this finding remains under investigation [86]. Anti-AQP4 antibodies were shown to mediate complement-dependent cytotoxicity (CDCC) in vitro [81]. In the frame of the relatively immunoprivileged condition of the CNS, complementary inhibitors are absent and this fact may account for the anti-AQP4 antibody-mediated CNS-specific immune response and tissue distraction. In this respect, NMOSD is primarily caused by damage to astrocytes whereas demyelination is secondary, in strike contrast to MS, a hypothesis that is further supported by neuropathological evidence [83].

In terms of clinical and radiological appearance, the localization of lesions in NMOSD is consistent in the regions where AQP4 is expressed at higher densities [79]. NMOSD may present clinically with Longitudinally Extensive Transverse Myelitis (LETM), optic neuritis, area postrema syndrome, acute brainstem syndrome and acute diencephalic syndrome. The episodes are often severe [68]. Lesions in NMOSD-associated myelitis typically affect the central grey matter of the spinal cord and extend over three contiguous vertebral bodies. Optic neuritis may involve both nerves simultaneously, typically affecting posterior 1/3 optic nerve segments and the anterior half of the optic chiasm [68]. Area postrema syndrome may manifest with nausea-vomiting and otherwise inexplicable hiccup and/or acute diencephalic syndrome with symptomatic narcolepsy [83]. The brain MRI lesions in NMOSD overall should not resemble MS. In this respect, juxtacortical U-fiber lesions, ovoid lesions adjacent and perpendicular to lateral ventricles, although not required for the diagnosis of MS, are considered highly indicative of MS and pose a red flag whenever diagnosis of NMOSD is considered [79].

Early diagnosis and treatment are crucial in order to decrease and/or delay the long-term accumulation of disability. Moreover, accurate diagnosis is of particular value due to the fact that DMTs typically indicated for MS often lead to disease exacerbation in patients with NMOSD [68]. Management of an acute relapse includes high dose intravenous steroids and plasma exchange in cases nonresponsive to steroids [81]. Long-term immunosuppression was suggested until recently, most commonly with mycophenolate mofetil or azathioprine [83]. In the recently developed “era of B-cell-depleting treatments”, rituximab, a chimeric anti-CD20 monoclonal antibody, is preferred due to a favourable safety profile, compared to classical immunosuppressants. However, a consensus with respect to the overall treatment duration and the frequency of the administration remains to be developed [68]. Complementary inhibitors, such as the monoclonal antibody eculizumab, were also recently associated with favourable outcomes in patients with NMOSD [79].

### 7.2. MOG Antibody Disease (MOGAD)

MOGAD is an inflammatory demyelinating disease of the CNS [87]. Due to the recent description of clinical syndromes associated with anti-MOG antibodies [82], MOGAD is not mentioned in the Orphanet classification under rare neuroinflammatory conditions of the CNS. However, two of the main phenotypes, representing diseases that exhibit significant clinical overlap with MOGAD, namely, ADEM and NMOSD [68], are considered rare neuroinflammatory diseases [48], and MOGAD most likely is expected to be similarly classified. Optic neuritis, often bilateral, and myelitis are other common manifestations [87]. MOGAD may manifest as either a monophasic or relapsing neuroinflammatory disease in the presence of anti-MOG antibodies in the patients’ serum, detected by the use of a cell-based assay [88]. MOG is a glycoprotein found on the myelin sheath, expressed exclusively in the CNS. It is located on the extracellular surface of myelin sheaths and on oligodendrocytes [87]. Its expression begins late in myelination. It is suggested that MOG may be a potential marker for oligodendrocyte maturation and the maintenance of myelin sheaths [89,90,91]. 

In the frame of MOGAD, transverse myelitis has been associated with accumulation of long-term disability [87]. With respect to optic neuritis, anti-MOG seropositive patients are at lower risk of further relapses and have better visual field outcomes compared to anti-AQP4 seropositive patients [87]. Brain MRI is abnormal in half of patients at onset [92]. Of those, the majority bears bilateral lesions and frequently exhibits infratentorial lesions. Lesion types highly indicative of MS, such as Dawson’s fingers, U-shaped and ovoid lesions, are rare and pose a red flag [87]. Contrary to MS and NMOSD-associated optic neuritis, in MOGAD-associated optic neuritis, the optic nerve appears more edematous, with anterior localization of the inflammation and usually sparing the chiasm [93].

MOG is a component of myelin. In contrast to AQP4, a molecule present in areas also outside the CNS, MOG is restricted to the CNS [89,90,91]. Although patients with anti-MOG antibodies exhibit demyelination as documented by CSF analysis, they also exhibit additional CSF characteristics, thus indicating that MOGAD is distinct from MS [82]. Moreover, patients with anti-MOG antibodies do not exhibit Glial Fibrillary Acidic Protein (GFAP) elevation in the CSF and are anti-AQP4-Ab seronegative even at the presence of an NMOSD phenotype, indicating that MOGAD is distinct from NMOSD [94]. Similarly, the pathology of CNS lesions in MOGAD verifies the unique nature of the disease: although some similarities to type-II MS pattern demyelinating lesions have been described, there is prominent complement deposition and preservation of pre-oligodendrocytes that do not express MOG [94,95,96,97,98].

Therapeutically, there are no controlled treatment trials in MOGAD; therefore, current treatment protocols tend to follow those for AQP4-NMO [87]. Treatment of relapses includes intravenous methylprednisolone for 3–5 days and plasma exchange as a second-line treatment. Long-term treatments with prednisolone per os, intravenous immunoglobulins, rituximab, mycophenolate mofetil, methotrexate or azathioprine have been shown to reduce annualized relapse rate in MOG-AD [99]. Treatment with interferon beta and glatiramer acetate has been shown to be ineffective [87].

### 7.3. Biomarkers in NMOSD and ADEM/MOGAD

Due to clinical overlap, the description of disease-specific antibodies for the diagnosis of NMOSD and MOGAD greatly facilitated the solution of diagnostic dilemmas. Up to 40% of patients with NMO, recurrent LETM or recurrent optic neuritis are anti-AQP4-seronegative upon disease onset and during the course of the disease [100] and anti-AQP4 antibodies are present in the serum of 70–90% of patients with NMOSD. Moreover, approximately one third of the patients with anti-MOG-Abs fulfil the current diagnostic criteria for NMOSD in terms of clinical presentation and/or radiology [101,102]. Unlike MS, OCBs are not common in NMOSD, and the CSF analysis may show mixed pleocytosis [68]. The laboratory assay with the highest sensitivity and specificity for the detection of anti-AQP4 antibodies is the cell-based assay, according to which the patients’ serum is tested for CDCC-related activity against transfected cells lines that express the conformational epitope of AQP4 in vitro. Anti-AQP4 antibody titres are significantly higher in blood compared to the CSF. This observation, together with the fact that in patients with NMOSD the hypermutated variable heavy chains of the B cells in the blood and the CSF are aligned, underlines the assumption that NMOSD is a primarily outside-in pathology [103,104]. 

Currently, there is no consensus on diagnostic criteria for MOGAD, with the exception of international recommendations on antibody testing for MOG encephalomyelitis [88]. According to these, testing for anti-MOG antibodies should be applied to selected cases with clinical features that fall under the MOGAD clinical and radiological spectrum. This is recommended in order to avoid false positive results and the rare AQP4-IgG/MOG-IgG “double-positive” test results that especially prompt for consideration [88]. Existing evidence indicates that the anti-MOG antibodies may be applied as a prognostic biomarker in MOGAD, but this assumption is less well-studied compared to the antibodies’ diagnostic value. Anti-MOG antibody titres typically are higher in relapse compared to remission, and they usually decrease upon monophasic disease [92]. In MOGAD, CSF analysis reveals pleocytosis in the majority of patients and elevated protein approximately in one third, whereas the presence of OCBs is rare [102].

The assumption that anti-MOG antibodies may be applied as a biomarker for long-term immunosuppressive or immunomodulating treatment in MOGAD remains under investigation [75]. This is in contrast to AQP4-IgG-positive NMOSD, which is typically associated with poor outcomes in terms of disability if left untreated, and on this basis, chronic immunosuppression is the norm [75]. MOGAD is associated with recurrent episodes to an extent of approximately 50% of adult patients [101,105]. However, these attacks are overall associated with more favourable prognosis compared to the anti-AQP4-antibody-seropositive patients [101,105]. However, debilitating episodes of recurrent optic neuritis and/or myelitis have also been described in MOGAD [101,102,105,106], and these reports prompt for careful selection of patients with MOGAD who may be considered eligible for long-term immunotherapy. Overall, children exhibit full recovery of the first attack more frequently than adult patients with MOGAD and the degree of recovery has been advocated as a factor to be taken under consideration when contemplating long-term immunotherapy [107,108]. Other factors that indicate towards immunotherapy are the recurring nature of the episodes; disease manifestations associated with poor outcomes, such as the chronic relapsing inflammatory optic neuropathy-like phenotype; and a persistent anti-MOG antibody beyond six months following an episode [75]. Typically, for children with full recovery of a first episode in the context of MOGAD that are anti-MOG-seronegative at a later follow-up, active observation with clinical and laboratory evaluation in the absence of chronic immunotherapy is suggested [75].

## 8. Conclusions

The paradigm of anti-MOG antibodies and their evolution in clinical practice underline the utility of a diagnostic biomarker in order to define a clinical entity as well as the significance of appropriate laboratory bioassays for proper evaluation and implementation of candidate biomarkers. The CNS as an organ poses limitations to this procedure, being relatively inaccessible and with a poor reflection of relevant pathological processes in the peripheral blood. With respect to rare demyelinating neuro-inflammatory disease, clinical and radiological heterogeneity signify the need for more accurate phenotype characterization, development and frequent update of diagnostic and management consensus guidelines and detailed, evidence-based definition of clinical outcomes. Rare MS variants are still diagnosed on the basis of their historical descriptions of clinical course, pathology and radiology. In spite of laboratory evidence, it remains unclear whether these entities actually fall under the MS spectrum. Moreover, newly described entities such as NMOSD and MOGAD may be accurately diagnosed on the basis of a well-characterized biomarker; however, universal guidelines regarding patient selection eligible for long-term immunotherapy and relevant treatment protocols are still lacking. Rare and less well-defined entities in particular require individualized approaches with respect to therapeutic decisions and long-term management. Large collaborations and network development with a reference to rare neuro-inflammatory disease are necessary in order to fully characterize the clinical, radiological, biological and pathological spectrum of these syndromes.

## Abbreviations

CNS	Central Nervous System
MS	Multiple Sclerosis
ADEM	Acute Disseminated Encephalomyelitis
NMOSDs	Neuromyelitis Optica Spectrum Disorders
MOG	Myelin Oligodendrocyte Glycoprotein
BCS	Baló Concentric Sclerosis
NMO	Neuromyelitis Optica
MOGAD	Anti-MOG antibody-mediated disease
DMTs	Disease Modifying Treatments
MRI	Magnetic resonance imaging
RRMS	Relapsing-remitting MS
OCBs	Oligoclonal bands
CSF	Cerebrospinal fluid
MDS	Myelinoclastic diffuse sclerosis
POMS	Pediatric-onset MS
DIT	Dissemination In Time
IgG	Immunoglobulin G
IVIG	IV immunoglobulin
anti-AQP4	Anti-aquaporin-4
AQP-4	Aquaporin-4
CDCC	Complement-dependent cytotoxicity
LETM	Longitudinally extensive transverse myelitis
GFAP	Glial Fibrillary Acidic Protein
MOGAD	MOG-Antibody Disease
